# Fetal Cardiac Timing Events Estimation From Doppler Ultrasound Signals Using Swarm Decomposition

**DOI:** 10.3389/fphys.2019.00789

**Published:** 2019-06-21

**Authors:** Saeed Alnuaimi, Shihab Jimaa, Yoshitaka Kimura, Georgios K. Apostolidis, Leontios J. Hadjileontiadis, Ahsan H. Khandoker

**Affiliations:** ^1^Department of Electrical and Computer Engineering, Khalifa University, Abu Dhabi, United Arab Emirates; ^2^Graduate School of Medicine, Tohoku University, Sendai, Japan; ^3^Department of Electrical and Computer Engineering, Aristotle University of Thessaloniki, Thessaloniki, Greece; ^4^Healthcare Engineering and Innovation Center, Department of Biomedical Engineering, Khalifa University, Abu Dhabi, United Arab Emirates

**Keywords:** doppler ultrasound, fetal assessment, fetal cardiac intervals, fetal heart rate, swarm decomposition

## Abstract

Perinatal morbidity and mortality can be reduced when any cardiac abnormalities during a pregnancy are diagnosed early. Doppler Ultrasound Signals (DUS) are often used to monitor the heart rate of a fetus and they can also be used to identify the timing events of fetal cardiac valve motions. This paper proposed a novel, non-invasive technique which can be used to identify the fetal cardiac timing events based upon the analysis of fetal DUS (based upon 66 normal subjects belonging to three differing age groups) which can later be used to estimate fetal cardiac intervals from a DUS signal. The foundation of this method is a novel decomposition method referred to as Swarm Decomposition (SWD) which makes it possible for the frequency contents of Doppler signals to be associated with cardiac valve motions. These motions include the opening (o) and closing (c) of Aortic (A) and Mitral (M) valves. When compared the SWD method results to the Empirical Mode Decomposition for the validation, the fetal cardiac timings were estimated successfully when isolating the constituent parts of analyzed DUS signals with reduced complexity compared to EMD method. Pulsed Doppler images are used in order to verify the estimated timings. Three fetal age groups were assessed in terms of their cardiac intervals: 16–29, 30–35, and 36–41 weeks. The time intervals (Systolic Time Interval, STI), (Isovolumic Relaxation Time, IRT), and (Pre-ejection Period, PEP) were found to change significantly (*p* < 0.05) across the three age groups. The evaluation of fetal cardiac performance can be enhanced, given that these findings can be leveraged as sensitive markers throughout the process.

## Introduction

Fetal well-being can be evaluated using a range of methods and Fetal Heart Rate (FHR) is one of the principal ones. Statistics reveal that each year one out of each 125 babies is born with a manner of congenital cardiac defect (American Heart Association, 2004). Fetal cardiac assessments are an essential tool in the early detection and diagnosis of cardiac diseases including types of Congenital Heart Diseases (CHDs) (Malcus, 2004). Without early detection and subsequent treatment, these diseases have the potential to threaten the life of a fetus. This is why early diagnosis is critical to reducing both perinatal morbidity and mortality while also providing various medical and economic benefits (Merz, 2004; Hameed and Sklansky, 2007). Fetal cardiac assessments are used to monitor the fetal heart rate and while it is a useful method it does not provide a fully comprehensive assessment of fetal cardiac activity. Valve motion timings can be measured and assessed using non-invasive methods such as Doppler Ultrasound Signal (DUS) or fetal echocardiography.

Of the two methods, fetal echocardiography is expensive and requires the abilities of skilled professionals which means it is reserved for particular cases. The DUS technique is also non-invasive but does not require as much skills or sophistication. DUS uses the Doppler shift of an ultrasound beam to reflect against the fetal heart’s moving valves, tracking the activity (Murata and Martin, 1974; Murata et al., 1978; Shakespeare et al., 2001). DUS has a key disadvantage, however, in that fetal movement can result in signal loss and misdiagnosis. At particular stages of the pregnancy, such as the early gestational age, the fetus moves a lot, and this can be problematic.

Doppler ultrasound signals are also used in clinical settings to assess flow velocities in different arteries. It is used for identifying intrauterine growth restriction among other risk conditions. Fetal growth restriction (FGR) is defined as the failure of the fetus to reach its genetically determined growth potential. FGR is a major determinant of perinatal and childhood morbidity and mortality, and is associated with the risk of chronic diseases in later life. Currently, fetal biometry and Doppler flow velocimetry are the major approaches used for FGR diagnosis. Gaccioli et al. (2018), The fetal weight (EFW) can be estimated from ultrasonic measurements of the fetus head size, abdominal circumference, and femur length. Furthermore, Doppler flow velocimetry provides crucial information on the resistance to blood flow in the fetoplacental unit and it features in several proposed FGR definitions (Gaccioli et al., 2018).

**FIGURE 1:**
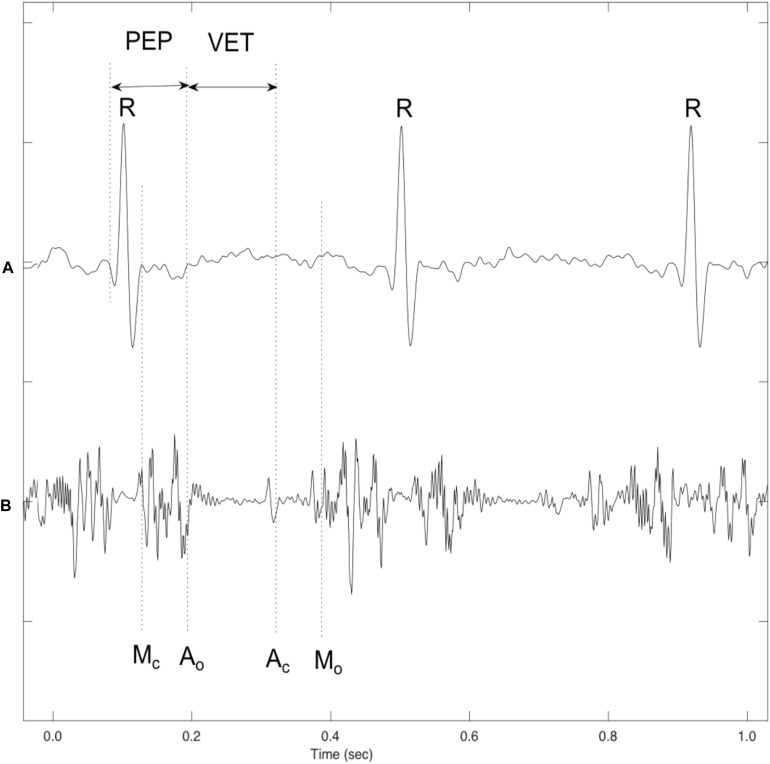
A sample of simultaneously recorded fetal ECG and Doppler ultrasound (DUS) signals: **(A)** Fetal ECG signal **(B)** Doppler ultrasound from fetal heart. Annotations showing the cardiac timing events of the opening and closing of the fetal heart’s valves with reference to the ECG signal. Mitral opening (M_o_), Aortic opening (A_o_), Mitral closing (M_c_), Aortic closing (A_c_), Pre-ejection period (PEP), and Ventricular ejection time (VET) (Khandoker et al., 2007).

Our paper addresses these challenges through the analysis of fetal cardiac Doppler signals used to assess valves motion. To identify a valve motion using this signal, the components which correspond to the motion of the valve will be separated from those components which are related to the movements of other organs, valves, or fetal cardiac wall (Marzbanrad et al., 2016). Several non-invasive techniques were proposed by several studies in the 1980s, offered in a bid to improve the measurements of systolic time intervals using the DUS signal and non-invasive abdominal Electrocardiogram (ECG) (Murata et al., 1978; Organ et al., 1980; Sampson, 1980). The signal received from the Non-directional (ND) channel and the Fetal Electrocardiogram (FECG) are presented in the example shown in Figure 1, which includes wall movements (Atrial wall contraction and Ventricle wall contraction) as well as the relative timings of the various heart valves Aortic closing (Ac), Aortic opening (Ao), Mitral closing (Mc), and Mitral opening (Mo). When Short Time Fourier Transform (STFT) analysis is applied to the DUS signals, it reveals that cardiac valve movements are linked to those components with a higher frequency band, while cardiac wall motion were linked with low-frequency components (Shakespeare et al., 2001). The multi-resolution wavelet analysis technique, meanwhile, was offered to decompose the DUS signal using variable spectral characteristic over time using the wavelet analysis signals (Khandoker et al., 2009a).

An alternative decomposition technique was proposed in the form of Empirical Mode Decomposition (EMD), which is leveraged in a bid to decompose non-linear and non-stationary time series. This technique has been applied to a range of fields including image, speech, and biomedical signal processing (Bouzid and Ellouze, 2004; Echeverria et al., 2010). The components which are linked to valve movements can be effectively separated using the EMD technique (Marzbanrad et al., 2013). Furthermore, the fetal cardiac cycles in the EMD method were segmented by using a reference ECG signal. In our paper the Swarm Decomposition Method is used to analyze fetal Doppler signals in a bid to identify the key fetal cardiac timing events. The main goal of using the SWD method is to identify the fetal cardiac timing events with reduced complexity compared to the Hybrid models (Marzbanrad et al., 2014). Moreover, using the SWD method could achieve an excellent segmentation of the fetal cardiac cycles without using any reference signal such as ECG signal. A review paper published by the authors assessed these recent methods which have been developed to monitor the fetal heart rate while affording a particular focus to identifying the event timing of fetal cardiac valve motions (Alnuaimi et al., 2017).

## Materials and Methods

The purpose of this section is to provide information about the collected DUS signals and to provide a background about the swarm decomposition (SWD) method.

### Subjects

The DUS signals were collected by a 1.5 MHz Ultrasonic Transducer 5700. Doppler ultrasound signals were recorded simultaneously from 66 pregnant women with normal single pregnancy at the gestational age of 16 to 41 (33 ± 6) weeks at Tohoku University Hospital, Japan. The extracted DUS signal components were compared in three age groups of early gestation age (16–29 weeks), mid gestation age (30–35 weeks) and late gestation (36–41 weeks), including 16, 21, and 29 fetuses in each age group, respectively. All recordings were 1 min length and sampled at 1 kHz with 16-bit resolution.

Fetal electrocardiogram signals were extracted from the composite abdominal signal using a method that combines the cancelation of the mother’s ECG signal and the Blind Source Separation (BSS) with the reference signal (Sato et al., 2007). M-mode and pulsed-wave Doppler were acquired simultaneously with DUS and FECG recordings, using convex 3.5 MHz of HITACHI ultrasound scanner, to verify the mitral and aortic valve opening and closing time obtained from the DUS signal. The study protocol was approved by Tohoku University Institutional Review Board and written informed consent was obtained from all participants.

The inclusion and exclusion criteria were considered in this study. In the inclusion criteria, signed on written consent form was provided, the age 20 years or older, the gestational age in the range of 24∼42 weeks, prenatal check-up results are urine protein (+) or less, urine sugar (+) or less, Blood pressure less than 140/90 mmHg and negative indirect Coombs test results. In the exclusion criteria, patients who were diagnosed as having an infectious disease (hepatitis B, hepatitis C, HIV, syphilis, HTLV-1, rubella, or chlamydia) at the time of registration based on the result of an infection test performed during the current pregnancy. Diagnosed with multiple pregnancy, abnormal pregnancy, pregnancy with an obstetric complication (e.g., gestational diabetes, gestational hypertension, uterine fibroids, and cervical cancer). Diagnosed with a serious medical disease or a mental illness, severe anemia (Hb 8.0 g/dl or less). Scheduled for Cesarean section (Oshio et al., 2018). Furthermore, Table 1 provides the essential maternal and fetal characteristics of the studied pregnancies in three gestational age groups such as the completed weeks of gestation at birth, mother age, the Estimated Fetal Body Weight (EFBW) and the Body Mass Index (BMI) (Oshio et al., 2018).

**TABLE 1:** Background of the essential maternal and fetal characteristics and measurement conditions in the three gestational groups.

	**Age**	**Gestational**	**EFBW**	**BMI**
	**(years)**	**weeks**	**(g)**	**(kg/m^2^)**
**Early group: 16 cases from 16 to 29 weeks of pregnancy**				
Mean	32	27	1180	22.57
Standard deviation	6.5	1.8	312.7	2.5
**Mid group: 21 cases from 30 to 35 weeks of pregnancy**				
Mean	37	34	2262	24.7
Standard deviation	5.3	1.5	188.1	3.9
**Late group: 29 cases from 36 to 41 weeks of pregnancy**				
Mean	33	39	3015	26.3
Standard deviation	5.6	1.3	190.4	4.4

*EFBW, Estimated Fetal Body Weight; BMI, Body Mass Index.*

The DUS component was segmented into cardiac cycle sections and then normalized. Segmentation was performed by detecting the R-peaks from the extracted SWD component and then using these R-peaks to segment the cardiac cycles. Each segment of the DUS component was taken from R-R intervals. It was then normalized by subtracting the mean and dividing by the standard deviation of the DUS component estimated over the segment as shown in Figure 4C. In order to detect the peak timings of both aortic and mitral valves motion events, the time durations from R wave within each RR interval chosen for each event were 0.05∼0.10 s for Ao and 0.14∼0.26 s for Ac. While for mitral valve’s relative timings, 0.00∼0.05 s for Mc and 0.26∼0.33 s for Mo were chosen in calculation (Khandoker et al., 2009b; Moosavi et al., 2017). The instantaneous energy of the SWD has been generated to compared with extracted SWD component.

### Swarm Decomposition (SWD) Method

Apostolidis and Hadjileontiadis (2017) were the first to use the concept of Swarm Filtering (SwF) as the basis of the signal decomposition method. This method offers a new perspective on this single-channel method which decomposes non-stationary signals over a set of functions – the constituent components of that signal which are localized in terms of both frequency and time. Those components do not have constant frequency or amplitude all the time. The concept has proven its worth in biomedical signal processing (Apostolidis and Hadjileontiadis, 2017). The Swarm intelligence (SI) based method is an atomic decomposition method where the non-stationary signal components are resulted a posteriory (Apostolidis and Hadjileontiadis, 2017). This has been done in a bid to use a rigid mathematical methodology rather than an empirical one while leveraging the advantages of EMD (Marzbanrad et al., 2014; Apostolidis and Hadjileontiadis, 2017). Speaking with more specificity, the SwF concept was applied (Marzbanrad et al., 2014) as the foundation of the decomposition method which is to be used, namely the swarm decomposition (SWD). SwF is based upon a swarming model wherein the processing is considered intuitively as a virtual swarm–prey hunting, making it a discrete time signal processing algorithm.

Furthermore, the swarming model consists of two types of interaction that realize the swarm–prey hunting. On one hand, the driving force ${\text{F}}_{\text{Dr},\text{i}}^{\text{n}}$ is the one the prey induces to every member of the swarm causing the hunting and the cohesion force ${\text{F}}_{\text{Coh},\text{i}}^{\text{n}}$ is induced among the members of the swarm, assuring the cohesion of the swarm (Gazi and Passino, 2003). This force has a dual nature; it can operate as attractive, keeping the members close to each other, or as repulsive, avoiding collisions. In SwF, the processing of a signal is inspired by the procedure where a swarm of birds hunts a prey. During the hunting, every member of the swarm updates its state (Apostolidis and Hadjileontiadis, 2017), i.e.,

$$v_{i}\,\left[n\right]=v_{i}\,\left[n-1\right]+\delta .\left(F_{\mathrm{𝐷𝑟},i}^{n}+F_{\mathrm{𝐶𝑜ℎ},i}^{n}\right),$$

$$p_{i}\,\left[n\right]=p_{i}\,\left[n-1\right]+\delta .v_{i}\,\left[n\right],$$

where δ is a parameter that controls the flexibility of the swarm. Particularly, δ determines the virtual time interval between two consecutive time steps. Using SwF systematically requires a relationship between particular responses and SwF parameters, extracted using a Genetic Algorithm (GA). The SWD is eventually realized through the iterative application of SwF. In each iteration an oscillatory mode sequence is achieved given that each SwF is properly parameterized (Apostolidis and Hadjileontiadis, 2017). The SWD is based on properly parameterizing SwF, so as to result in Oscillatory Components (OCs) of a multi-component input signal, s[n], ∀ *n* = 0, …, L—1. Specifically, the SWD consists of an iterative execution of a sifting-like process, where at every iteration the dominant OC is initially guessed, and then this OC is obtained by consecutive applications of SwF (Apostolidis and Hadjileontiadis, 2017).

**FIGURE 2:**
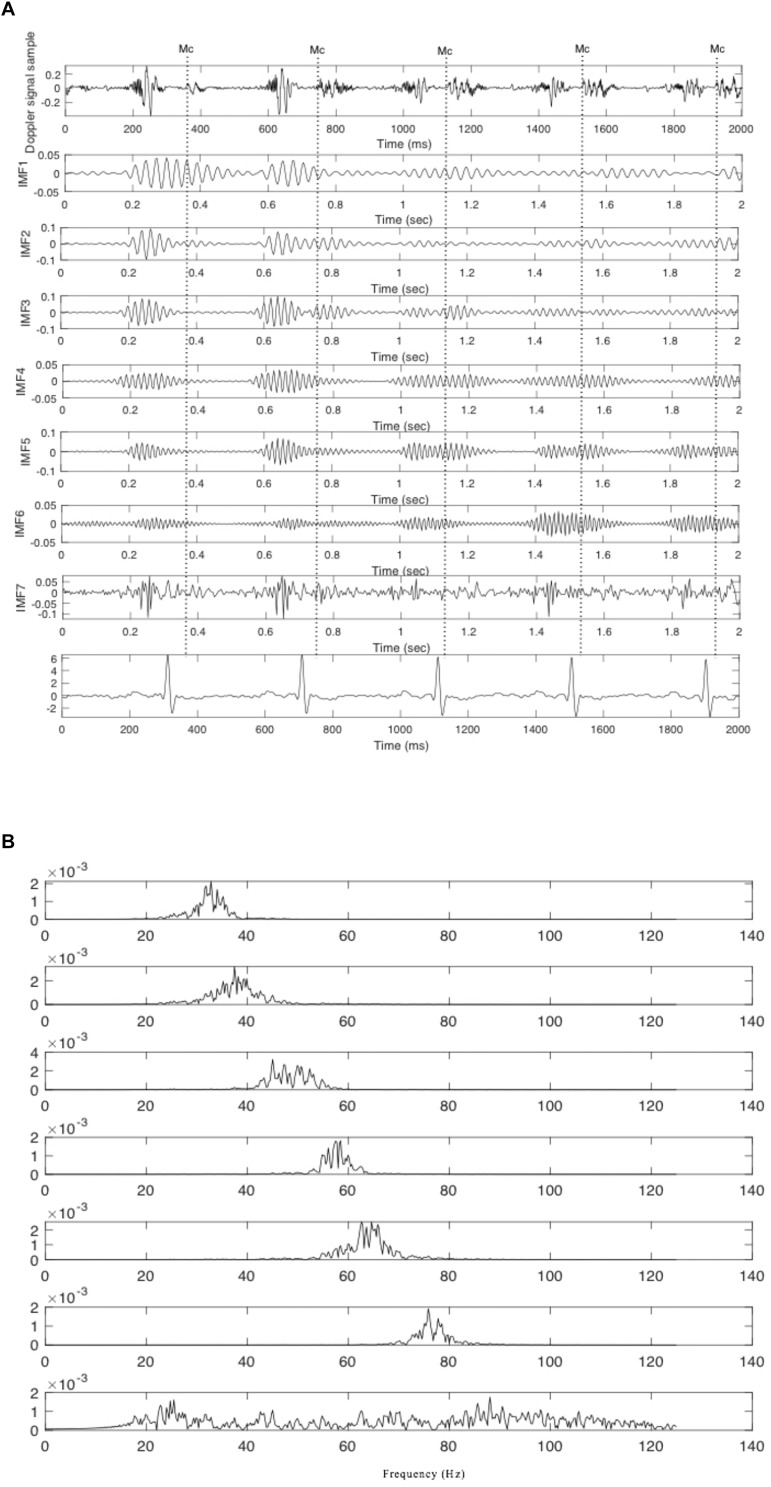
The extracted components from the fetal cardiac Doppler signal using SWD. **(A)** Time domain and **(B)** Frequency domain.

**FIGURE 3:**
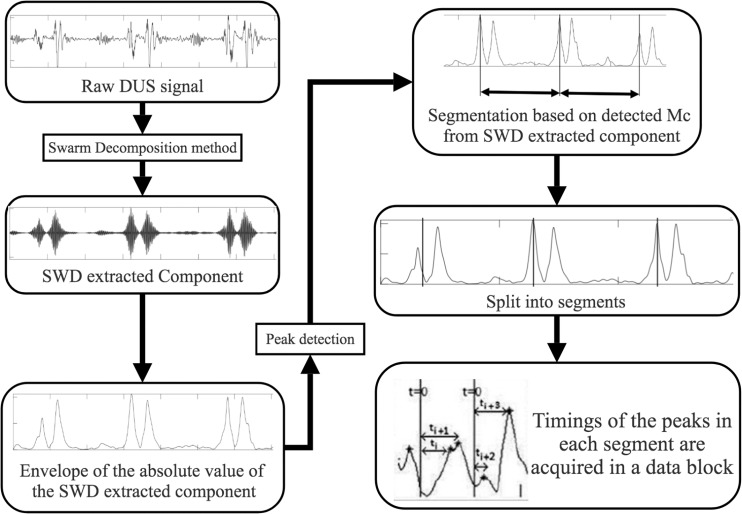
Cardiac events identification using SWD approach block diagram.

When using SwF, the route of a prey represents the input signal while the processing is treated as a virtual swarm–prey hunting. The output is found as a value which is taken from the swarm’s trajectory. Definitions for the prey, the forces, and the swarm cause the interactivity between the hunting participants and make up the swarming model. Specific swarm behaviors are brought about by adjustments made to the model parameters, such as SwF responses. A reinforcement learning that uses GA can be applied (Apostolidis and Hadjileontiadis, 2017) in a bid to uncover the relationships that are shared between the filter response and model parameters. Non-linear and non-stationary signals can be analyzed using SWD, a data-driven algorithm that does not require prior information about specific components to decompose the Doppler signal using a natural approach. A comprehensive explanation of the SWD method can be found in Apostolidis and Hadjileontiadis (2017). Their paper therefore proposes that SWD is applied to the DUS signal in order to decompose it over a series of specific functions which differ in terms of frequency bands. These extracted functions will be used later on to identify and estimate the fetal cardiac timing events. The estimated cardiac intervals were also analyzed by Kruskal–Wallis test to investigate their changes during pregnancy. Figure 2 provides an example of SWD being applied to DUS data. Furthermore, it can be seen from Figure 2 that the fetal cardiac cycles were been segmented using the extracted SWD components without using any reference signal such as ECG signal. The sample procedure for detecting a cardiac event is shown in Figure 3. In the next sections, the peaks of the envelope of the first component provide the features for the identification of the fetal cardiac events.

## Results

**FIGURE 4:**
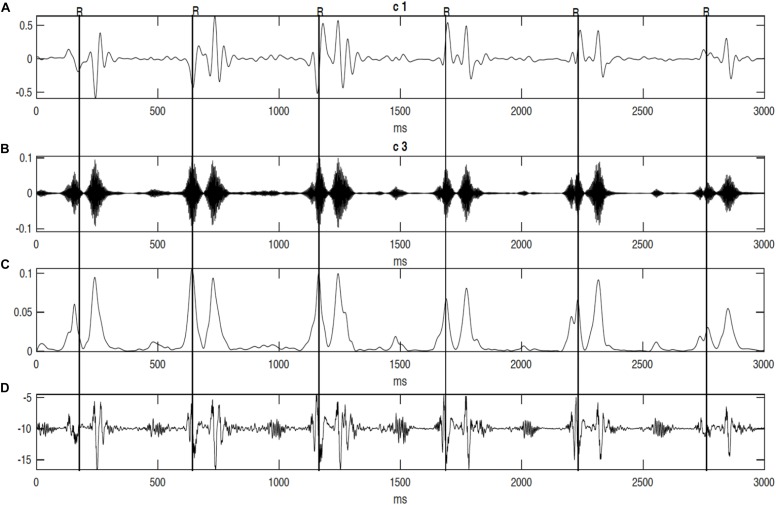
The decomposition of the DUS signal using swarm decomposition. **(A)** First SWD extracted component. **(B)** Second SWD extracted component. **(C)** The envelope of the absolute value of the Second SWD extracted component **(B)** and its segments divided by vertical lines. **(D)** Doppler ultrasound (DUS) signal.

**FIGURE 5:**
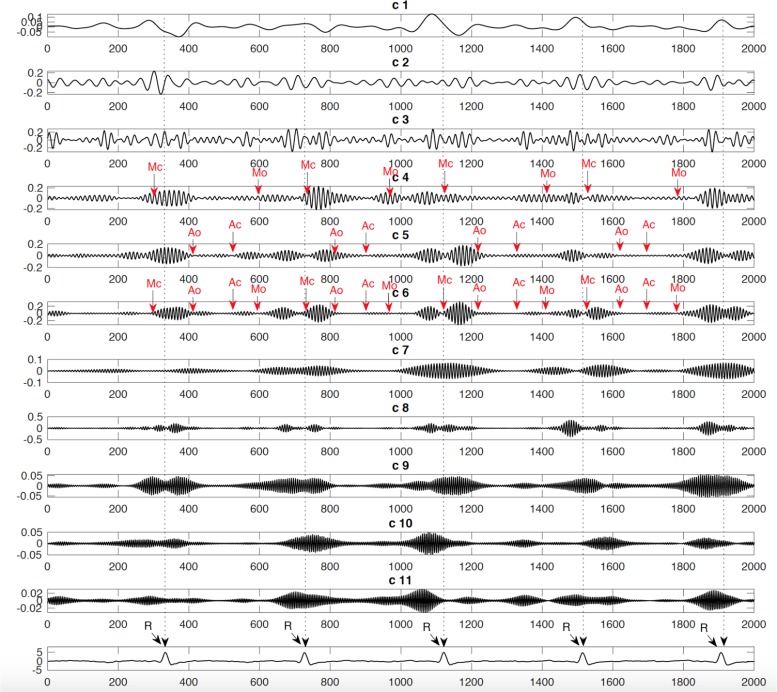
Example of identified events: Mitral opening and closing (Mo and Mc) and Aortic opening and closing (Ao and Ac) on the extracted components from the fetal Doppler signal using the Swarm decomposition.

Results demonstrated that the application of swarm decomposition brings about the separation of the component that is linked to valve movements. The peaks which relate to cardiac events can be differentiated as demonstrated in Figure 4. The derivative of the SWD component shown in Figure 4C was used to identify the peak of each DUS component segment. In some cases, it was possible to easily identify events. Figure 5 shows an example of the identified mitral opening and closing (Mo and Mc) and aortic opening and closing (Ao and Ac) on the extracted components from the fetal Doppler signal using the Swarm decomposition. There are some cases, however, where this was more complicated and only certain events were registered. The results of pairwise comparison indicate that except for ICT and VET, all intervals of the age group 36–41 are significantly different from previous ages. Table 2 outlines the mean duration and the standard error corresponding to the estimated cardiac events (for example, cardiac valves opening and closing timings from R peak of FECG namely R-Ac, R-Ao, R-Mc, R-Mo within each RR interval). In order to have an excellent SWD decomposition of the DUS signal, a good quality normal DUS signals have been selected for this purpose. Furthermore, Table 2 provides information about the mean and the standard error of the identified cardiac intervals for example ICT (isovolumic contraction time), IRT (isovolumic relaxation time), PEP (pre-ejection period), STI (systolic time interval), and VET (ventricular ejection time) in three different age groups.

**TABLE 2:** Mean ± standard error (SE) of the average time intervals (ms) between r-peak and fetal cardiac valves motions over 66 normal fetuses versus three different age groups and the mean and se values of the ICT (Isovolumic Contraction Time), IRT (Isovolumic Relaxation Time), PEP (Pre-ejection Period), STI (Systolic Time Interval), and VET (Ventricular Ejection Time) for 66 normal fetuses in three different age groups.

	**Age group 16–29**	**Age group 30–35**	**Age group 36–41**
**Intervals**	**(16 fetuses)**	**(21 fetuses)**	**(29 fetuses)**
	**Mean ±**	**Mean ±**	**Mean ±**
	**standard error**	**standard error**	**standard error**
R-R (ms)	404.56 ± 11.97	422.56 ± 7.54	421.98 ± 6.8
R-Mc (ms)	14.51 ± 1.27	15.89 ± 1	15.17 ± 0.76
R-Ao (ms)	59.4 ± 3.86	51.7 ± 1.02	53.2 ± 0.84
R-Ac (ms)	198.08 ± 3.72	196.19 ± 2.38	187.61 ± 2.87
R-Mo (ms)	275.57 ± 3.65	270.97 ± 1.52	266.39 ± 1.5
ICT (ms)	44.9 ± 3.1	35.8 ± 1.2	38 ± 1
IRT (ms)	77.5 ± 2.18	74.8 ± 2.9	78.8 ± 3.2
PEP (ms)	69.9 ± 3.1	60.8 ± 1.2	63 ± 1
STI (ms)	208.6 ± 3.19	205.3 ± 2.7	197.4 ± 3
VET (ms)	138.7 ± 3.36	144.5 ± 2.7	134.4 ± 3.3

*The total number of beats (*N* = 2258) in age group 16–29, (*N* = 2860) in age group 30–35, and (*N* = 4013) in age group 36–41.*

**FIGURE 6:**
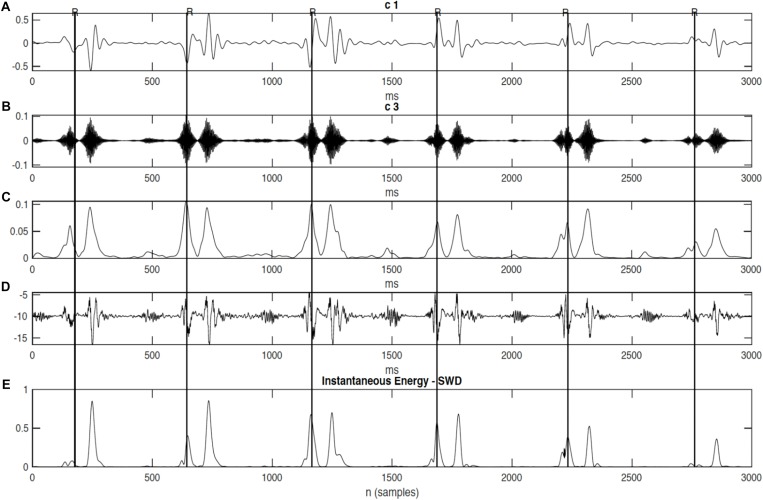
The decomposition of the DUS signal using swarm decomposition. **(A)** First SWD extracted component. **(B)** Second SWD extracted component. **(C)** The envelope of the absolute value of the Second SWD extracted component **(B)** and its segments divided by vertical lines. **(D)** Doppler ultrasound (DUS) signal, and **(E)** the instantaneous energy of the SWD.

**FIGURE 7:**
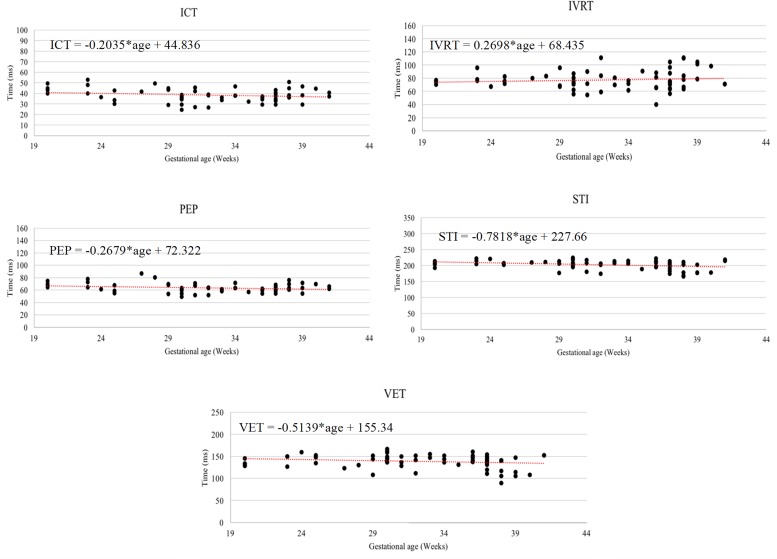
The Relationship between the following fetal cardiac intervals, VET (ventricular ejection time), PEP (pre-ejection period), STI (systolic time interval), and IRT (isovolumic relaxation time) with gestational age.

It can be seen from Figure 6, that R-peaks are clearly identified in Figure 6E and it matches the envelope of the absolute value of the Second SWD extracted component Figure 6C. The identified fetal cardiac intervals VET (Ventricular ejection time), PEP (Pre-ejection period), STI (Systolic time interval), and IRT (Isovolumic relaxation time) for the fetuses were all compared against the three different age groups by non-parametric statistical analysis as shown in Figure 7. Analysis of variance (ANOVA) test was used to compare the identified fetal cardiac intervals for the fetuses for early, mid and late age groups. *P*-value of 0.05 was chosen as the level of significance. Tables 3, 4 show the results of Kruskal–Wallis test (*p*-Values), mean and standard error of the timings for each age group, as well as their pair-wise *post hoc* multiple comparison with the Mann– Whitney–Wilcoxon method. As shown in Table 5, the results were compared with the previous EMD method, the comparison verified the successful identification of the events.

**TABLE 3:** *P*-value and correlation results for comparison of identified fetal cardiac intervals for the fetuses in Early gestation (20–29 weeks), Mid gestation (30–35 weeks), and Late gestation (36–41 weeks), using the Kruskal–Wallis test.

**Intervals**	***P*-value**	**Correlation (r)**	
		**Early to mid**	**Mid to late**
ICT (ms)	0.076280	0.29	−0.12
IRT (ms)	0.03191	0.40	−0.21
PEP (ms)	0.008	0.29	−0.12
STI (ms)	0.010396	0.60	−0.37
VET (ms)	0.132719	0.38	−0.03

**TABLE 4:** Results of *post hoc* multiple comparison by Mann–Whitney–Wilcoxon method (*P*-values).

**Intervals**	**16–29 vs. 30–35**	**16–29 vs. 36–41**	**30–35 vs. 36–41**
IRT	0.0045	0.1495	0.0266
PEP	0.0055	0.0319	0.0007
STI	0.5916	0.0020	0.0073

**TABLE 5:** Comparison between the mean ± standard error (SE) (ms) of the fetal cardiac timings for different age groups using both the EMD and SWD methods.

	**Mean ± standard error**		**Mean ± standard error**		**Mean ± standard error**	
**Interval**	**age group 16–29**		**age group 30–35**		**age group 36–41**	
	**EMD**	**SWD**	**EMD**	**SWD**	**EMD**	**SWD**
ICT	36.4 ± 2.6	44.9 ± 3.1	35.6 ± 2.7	35.8 ± 1.2	37.7 ± 3.4	38.0 ± 1.0
IRT	73.0 ± 4.6	77.5 ± 2.1	69.7 ± 4.5	74.8 ± 2.9	72.2 ± 4.9	78.8 ± 3.2
PEP	61.7 ± 4.8	69.9 ± 3.1	59.9 ± 5.2	60.8 ± 1.2	64.0 ± 4.0	63.0 ± 1.0
STI	213.9 ± 5.2	208.6 ± 3.1	214.0 ± 7.1	205.3 ± 2.7	218.2 ± 7.1	197.4 ± 3.0
VET	152.2 ± 3.7	138.7 ± 3.36	154.2 ± 6.9	144.5 ± 2.7	154.2 ± 7.7	134.4 ± 3.3

**FIGURE 8:**
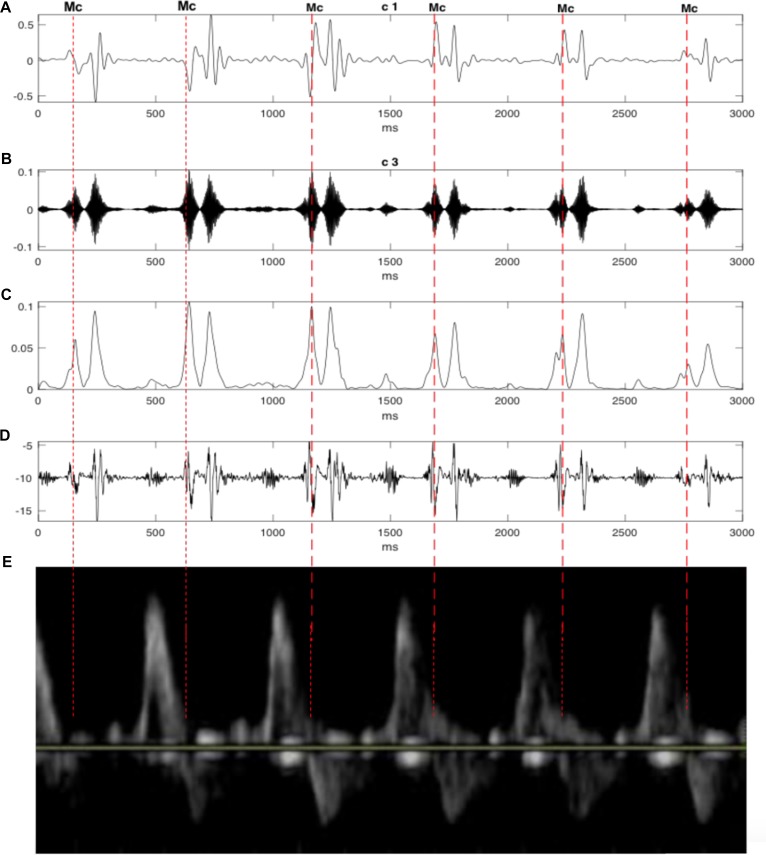
The decomposition of the DUS signal using swarm decomposition. **(A)** First SWD extracted component. **(B)** Second SWD extracted component. **(C)** The envelope of the absolute value of the Second SWD extracted component **(B)** and its segments divided by vertical lines. **(D)** Doppler ultrasound (DUS) signal. **(E)** The pulsed-wave Doppler signals during the opening and closing time of the Mitral and aortic valves.

**FIGURE 9:**
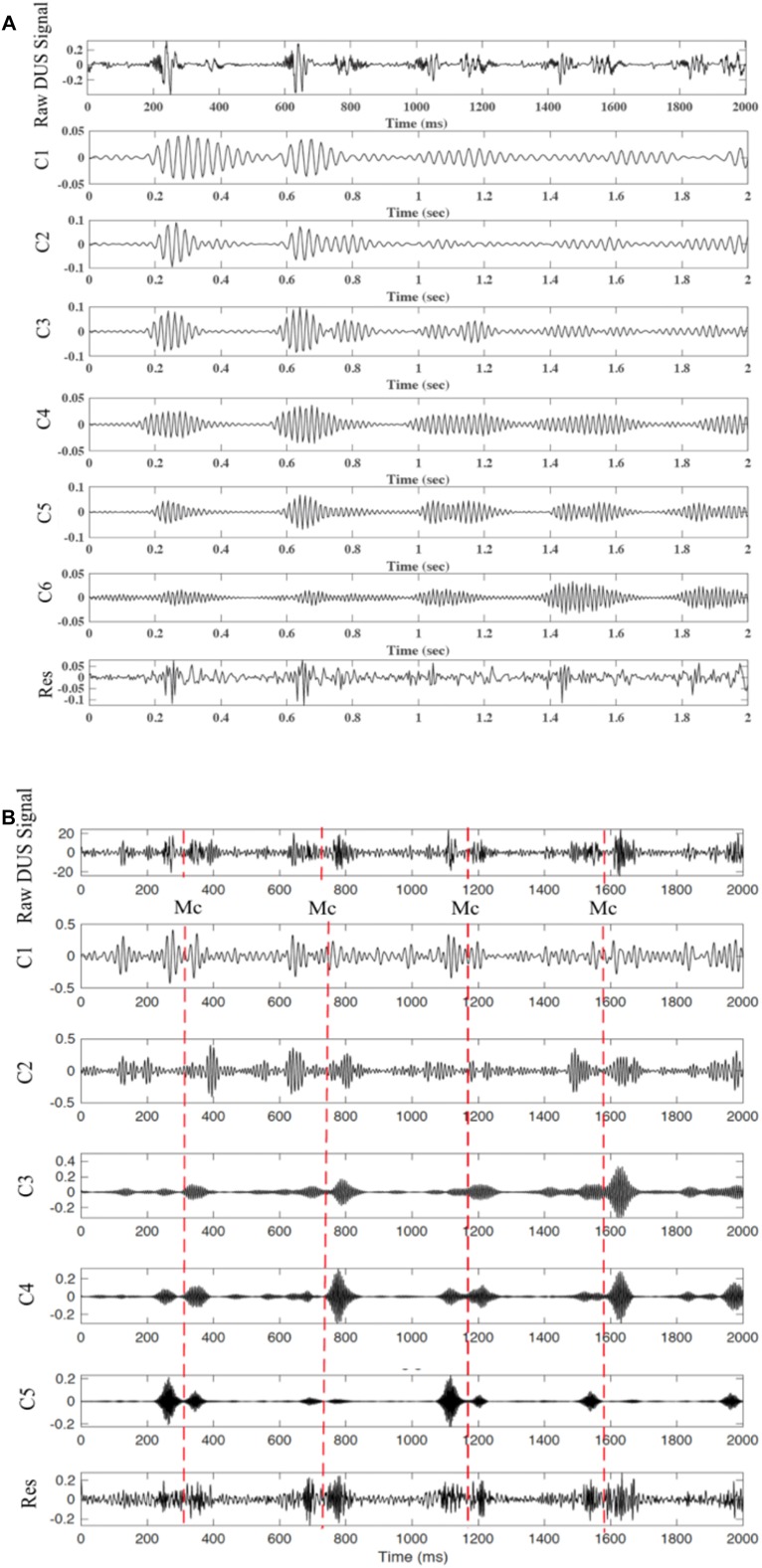
Decomposition of the DUS signal using SWD. **(A)** extracted components from a good quality DUS signal. **(B)** Extracted components from a bad quality DUS signal.

In order to evaluate the results, the timings of opening and closure of the valves were verified by the pulsed-wave Doppler images as shown in Figure 8. It visualizes the direction and the characteristics of the blood flow through the valves. In this technique, the aortic blood flow Doppler waveform is recorded from the long axis of the five-chamber view of the heart. The M-mode cursor is placed perpendicular to the Interventricular septum at the level of the mitral valve to examine end-systole and end-diastole (closure of atrioventricular valves). Furthermore, the swarm decomposition technique has been tested on different Doppler signal qualities. It can be seen from Figure 9, that both bad and good quality DUS signals decomposed using the swarm decomposition, the decomposition process for both signals achieved an excellent extraction of the fetal cardiac intervals.

## Discussion

Studying “normal” pregnancies leads to a better understating on diagnosing abnormal conditions prenatally, it may be possible to reduce perinatal morbidity and mortality. Furthermore, it provides tremendous medical, psychological, and economical benefits (Hameed and Sklansky, 2007). Previous studies have used the DUS signal to estimate various time intervals of cardiac events. Several methods were used such as short-term Fourier transform (STFT), digital filtering, wavelet analysis, and empirical mode decomposition (Koga et al., 2001b; Shakespeare et al., 2001; Marzbanrad et al., 2014). Shakespeare et al. (2001) used short-time Fourier transform to interpret the DUS signals from a fetal heart and conducted the identification of valve and wall motions which were observed in the Doppler signal, as well as the way that these fetal heart events were related. An automated estimation of fetal cardiac intervals from a DUS signal was proposed by Marzbanrad et al. (2014), wherein the signal is based upon a unique combination of hybrid support vector machines and empirical mode decomposition (EMD)—hidden Markov models (SVM/HMM).

Given that the fetal DUS signal is non-linear and non-stationary, wide changes are noted on a beat-to-beat basis in terms of the signal content and special characteristics. Inaccurate results are produced when fixed parameters are used, such as wavelet parameters for the whole signal or cut-off frequency for filtering techniques. This is why Swarm Decomposition – a data-driven method – is far more suitable. Initially developed for decomposing non-stationary and nonlinear signals (including EEG signals, Apostolidis and Hadjileontiadis, 2017), swarm decomposition has not been previously used in this way before. The observed results demonstrate that the component linked to valve movements can be practically separated through the application of swarm decomposition, and the peaks that correspond to specific cardiac events can be divided. The time intervals relating to the opening and closing of the heart’s valves can be matched when comparing the estimated timings of previous methods like EMD (Marzbanrad et al., 2016).

Without having FECG as a segmentation reference, it would have been very challenging to estimate the timing of cardiac events. Within this study, the R-wave position in the SWD-extracted element was used to segment the signal into different cardiac cycles without using FECG as a reference in comparison to the approach employed in Marzbanrad et al. (2016). Continuous segmentation and beat-to beat identification of cardiac intervals is provided by the results of this method and have important clinical uses. The study also investigated the close ties between the gestational age and the cardiac intervals. SIT was identified as the time interval that changes the most according to age, as revealed by the results obtained in Marzbanrad et al. (2014). According to reports by Koga et al. (2001a) ICT, on the other hand, was more stable during pregnancy. PEP increases along with gestational age, as revealed in a recent study by Mensah-Brown et al. (2010) This study uses comparison to discover that PEP decreases slightly from the 16–29 age group to the 30–35, before witnessing a significant increase to the 36–41 age group.

The results in Marzbanrad et al. (2014) revealed that except for EDT (electromechanical time delay) and ICT, each interval of the 36–41 age group are different when compared to previous ages. STI, for example, does not exhibit any significant change from the 16–29 to 30–35 age groups, but rather increases sharply as the final weeks of pregnancy approach. The late stage of the pregnancy also exhibits a trend of changes in PEP. This means that the final weeks of pregnancy are the most critical. The IRT interval timings reported in Kikallio et al. (2005) were shorter than those in this study and the proposed study (Marzbanrad et al., 2014). One reason for this is posited by the authors in Marzbanrad et al. (2014), who explain this may be because the age of the fetuses which were assessed in Kikallio et al. (2005) ranged from 6 to 10 weeks of gestation, while the average age of the fetuses analyzed as part of this study was 31 weeks. As the fetal heart develops further, there is a change to cardiac time intervals and cardiac function. This developmental change could be responsible for the difference. In human fetus, fetal ECG measurement was increasingly difficult from 30 to 33 weeks of gestation during third trimester because the insulating effects of vernix caseosa dampened the conduction of electrical potentials (Hoath et al., 2001). In our study, the fetal cardiac timing events were estimated from the DUS signal which reflects the mechanical activity of the fetus heart. The insulating effects of vernix does not affect the fetal DUS signals. The proposed swarm technique has a key advantage in that it permits for the efficient decomposition of a DUS signal into a series of components which retain and preserve physical meaning, similar to alternative decomposition techniques like Empirical mode decomposition (Marzbanrad et al., 2014). While the time complexity of swarm decomposition can be resolved by sampling the input signal, it is not appropriate to be used for real-time applications, making this a key disadvantage of the swarm decomposition method.

The quality of the DUS signal is usually affected by noise and also depends on other factors such as the fetus-transducer positioning and the amniotic fluid. Although the DUS quality assessment has been previously investigated, it was only targeted to enhance the FHR monitoring (Magenes et al., 2001; Stroux and Clifford, 2014). Marzbanrad et al. (2015) showed that the DUS quality can also be assessed in more detail, based on its reliability for valve motion identification. Furthermore, the swarm decomposition technique has been tested on different Doppler signal qualities as shown in Figure 9. Both types of the DUS signals achieved an excellent extraction of the fetal cardiac intervals. The SWD has a wider scope of potential use than those posited in this work. SWD can also be applied to a range of signal processing problems such as complexity reduction, event detection, and filtering. The SWD applications can also be extended in order to process higher dimension signals such as videos and images. Video processing, for example, has benefited from the application of the concepts explored in this work for interesting event detection (Kaltsa et al., 2015; Apostolidis and Hadjileontiadis, 2017). Work conducted in the future will include a quantitative comparison for pulsed wave Doppler image-based valve motion timings.

## Conclusion

The critical importance of clinical fetal heart rate monitoring and what it can achieve is presented in this paper. It also identifies DUS signals as being noisy, non-linear, non-stationary and varying according from one beat to another. This meant that a new method for fetal DUS signal analysis – which leverages the potential of swarm intelligence – was offered. An explanation of swarm decomposition was outlined, exploring this data-driven decomposition method that can produce a range of sequences which represent the temporal frequency characteristics of a non-stationary signal. The SWD method means that the frequency contents of fetal Doppler signals can be associated directly with the closing and opening of the aortic and mitral heart valves, improving the estimation of cardiac intervals. Pulsed Doppler images were used to verify the identified timings. What’s more, the STI, IRT, PEP, and VET fetal cardiac intervals were estimated with the SWD method. An analysis of changes of cardiac intervals among growing gestational age groups was also offered. Results demonstrated that from early to late gestational stages, the fetal cardiac intervals can vary significantly. In terms of next steps, the next is to widen the scope of the analysis in order to accommodate for abnormal fetal cardiac Doppler signal samples.

## Author Contributions

SA designed the study under the supervision of AK, SJ, and LH. YK supervised the data collection. GA developed the SWD tool. SA was responsible for analysis, running statistics, under the supervision of AK and SJ. SA wrote the manuscript and interpreted the results in consultation with AK and SJ. SA, AK, and SJ revised the final version of the manuscript.

## Conflict of Interest Statement

The authors declare that the research was conducted in the absence of any commercial or financial relationships that could be construed as a potential conflict of interest.
